# Supplementary material for: The shared frameshift mutation landscape of microsatellite-unstable cancers suggests immunoediting during tumor evolution
Source: Nat Commun. 2020 Sep 21;11:4740. doi: 10.1038/s41467-020-18514-5 (PMC7506541; doi:10.1038/s41467-020-18514-5)
Supplement: Supplementary file 1 — Supplementary Information [file 41467_2020_18514_MOESM1_ESM.docx]

Supplementary Information

# The shared frameshift mutation landscape of microsatellite-unstable cancers suggests immunoediting during tumor evolution

Authors:

Alexej Ballhausen^1-3,$^, Moritz Jakob Przybilla^1-3,$^, Michael Jendrusch^1-3,$^, Saskia Haupt^4^, Elisabeth Pfaffendorf^1-3^, Florian Seidler^1-3^, Johannes Witt^1-3^, Alejandro Hernandez Sanchez^1-3^, Katharina Urban^1-3^, Markus Draxlbauer^1-3^, Sonja Krausert^1-3^, Aysel Ahadova^1-3^, Martin Simon Kalteis^1-3^, Pauline L. Pfuderer^1-3^, Daniel Heid^1-3^, Damian Stichel^1,5^, Johannes Gebert^1-3^, Maria Bonsack^6-8^, Sarah Schott^9^, Hendrik Bläker^10^, Toni Seppälä^11^, Jukka-Pekka Mecklin^12^, Sanne Ten Broeke^13^, Maartje Nielsen^13^, Vincent Heuveline^4^, Julia Krzykalla^14^, Axel Benner^14^, Angelika Beate Riemer^6,7^, Magnus von Knebel Doeberitz^1-3^, Matthias Kloor^1-3*^

$ These authors contributed equally.

Affiliations:

1 Department of Applied Tumor Biology, Institute of Pathology, University of Heidelberg, Heidelberg, Germany.

2 Collaboration Unit Applied Tumor Biology, German Cancer Research Center (DKFZ), Heidelberg, Germany.

3 Molecular Medicine Partnership Unit (MMPU), Heidelberg University Hospital and EMBL Heidelberg, Germany.

4 Engineering Mathematics and Computing Lab (EMCL), Interdisciplinary Center for Scientific Computing (IWR), Heidelberg University, Heidelberg, Germany

5 Clinical Cooperation Unit Neuropathology, German Cancer Research Center (DKFZ), Heidelberg, Germany

6 Immunotherapy and Immunoprevention, German Cancer Research Center (DKFZ), Heidelberg, Germany

7 Molecular Vaccine Design, German Center for Infection Research (DZIF), partner site Heidelberg, Heidelberg, Germany

8 Faculty of Biosciences, Heidelberg University, Heidelberg, Germany

9 Department of Obstetrics and Gynecology, University Hospital Heidelberg, Heidelberg, Germany.

10 Institute of Pathology, University Hospital Leipzig, Leipzig, Germany.

11 Department of Gastrointestinal Surgery, Helsinki University Hospital and University of Helsinki, Helsinki, Finland

12 Department of Education and Research, Central Finland Central Hospital, Jyväskylä, Finland, and Sports and Health Sciences, University of Jyväskylä, Jyväskylä, Finland

13 Department of Clinical Genetics, Leiden University Medical Center, Leiden, The Netherlands

14 Division of Biostatistics, German Cancer Research Center (DKFZ), Heidelberg, Germany

* Corresponding author

SUPPLEMENTARY FIGURES

**Supplementary Figure 1. Concept and validation of ReFrame**.  (A) A10 cMS of the *TGFBR2* gene, including amino acid translation of wild type, *m1* (magenta) and *m2* (green) alleles. (B) Peak profile obtained from colonic normal tissue with a wild type *TGFBR2* A10 allele (upper panel). For comparison, the peak profile of matching tumor tissue, carrying *m1* and *m2* mutations is shown (lower panel). Pie charts illustrate allele frequency after removal of stutter bands by ReFrame. (C) Stutter ratios of raw data normal tissues are plotted against cMS repeat length. (D) MSI cell lines with known mutation status of a defined cMS (NDUFC2, LS180: *m1*/wt, HT29: wt) were mixed to compare experimental ReFrame results (black dots) with theoretically expected values (blue line). Three representative peak patterns (raw data prior to stutter band removal) are shown the right panel.

**Supplementary Figure 2.** **Mutation frequencies of cMS in MSI CRC and EC.** The relative frequency of mutant alleles analyzed by the qMSI algorithm is shown for 41 cMS (rows) in all CRC and EC tumor samples (columns). using ReFrame. cMS were sorted alphabetically. Dark blue represents high mutation frequency, whereas pale blue represents low mutation frequency. Black boxes indicate missing data points. cMS were analyzed for both CRC and EC and are depicted separately for each tumor type (left panel: CRC, right panel: EC). Source data are provided as a Source Data file.

**
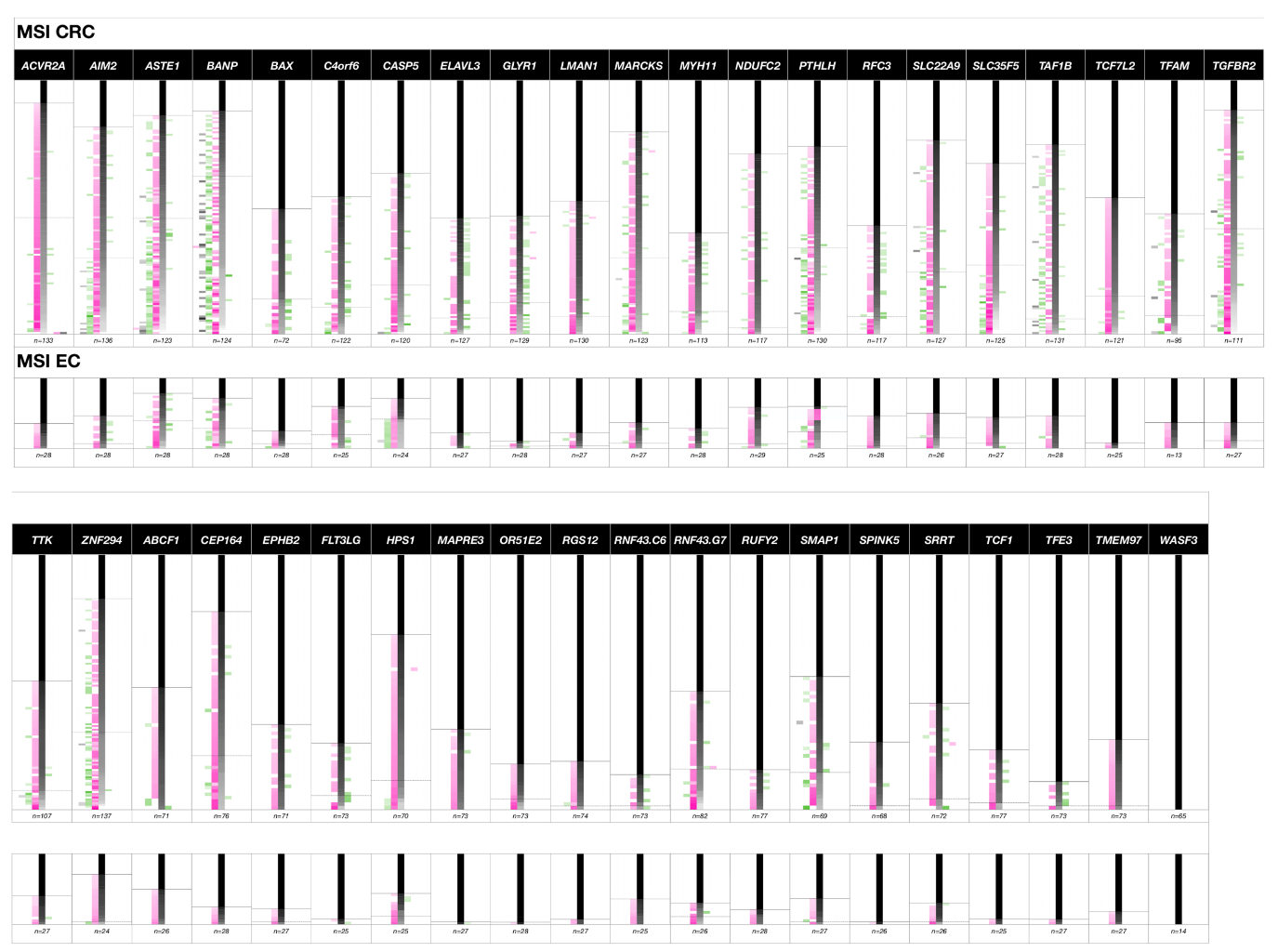
**

**Supplementary Figure 3. CMS mutation patterns based on ReFrame analysis.** The detailed mutational patterns of all 41 analyzed cMS are represented with their respective frequency of mutation for all possible resulting frameshift mutations in MSI CRC and EC. Each row constitutes one analyzed tumor sample with its related allele ratios. The number of samples analyzed for a certain candidate is indicated below for each candidate. Since wt, *m3* and *p3* mutations do not result in translational frameshifts, they are shown in black. In contrast, *m1*, *m4* and *p2* mutations (magenta) and *m2*, *p1*, *p4* mutations (green) are either resulting in a frameshift peptide arising from a one base pair or two base pair deletion reading frame, respectively. The column intensities represent calculated ratios from white (0%) to the respective color of the column (100%). All samples are sorted according to their wild-type proportion. The annotated solid lines show the end of the non-mutated tumor samples while the dotted lines mark the beginning of tumors that are mutated in more than 50 % of cases, associated with biallelic hits within the respective sample. Some candidates which were analyzed in CRC were not analyzed for EC (white spaces). Source data are provided as a Source Data file.

**Supplementary Figure 4.** **Epitope predictions in HLA-A*02:01.** The figures display the predicted epitopes in HLA-A*02:01, the most frequent HLA-type in the western world, for the M1 and M2 frameshift peptides of all 41 cMS candidates over the length of the respective peptide. High-affinity (IC_50_ < 50 nM), low-affinity (IC_50_ < 500 nM), and very low-affinity epitopes (IC_50_ < 5000 nM), are shown for the M1 frameshift peptides (left, magenta) and the M2 frameshift peptides (right, green). All candidates are sorted in alphabetical order with their respective frequency of mutation according to the results of the ReFrame algorithm. The grey field represents background and indicates C-terminal ends of the respective frameshift peptides.


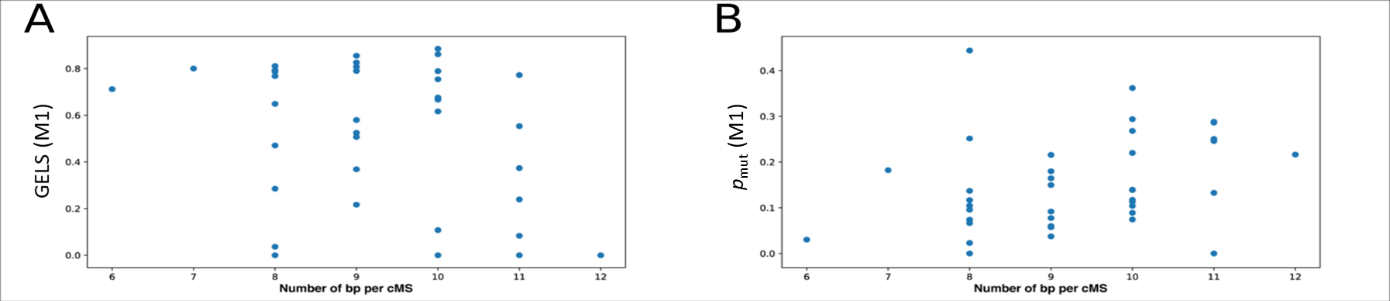


**Supplementary Figure 5. GELS and mutation frequency** *p*_mut_ **in dependency on cMS length.** (**A**) All cMS which were analyzed using ReFrame, thus having a calculated IRS, are depicted according to their number of base pairs on the x-axis and their GELS on the y-axis. The GELS is shown for the M1 frame only. (**B**) All cMS which were analyzed using ReFrame are depicted according to their number of base pairs and their mutational frequency on the y-axis. The mutational frequency is shown for the M1 frame only.

**Supplementary Figure 6. HLA binding affinity predictions for 10 frameshift peptides.** (**A**) HLA binding predictions for the M1 frameshift peptide of TGFBR2 is shown for the HLA supertype representatives. HLA binding prediction for the respective HLA type over the length of the neoantigen is shown in each column. Summarized hot spots of predicted HLA binding are shown at the top of each figure. For each candidate, the three different parts of the figure represent peptides with regard to their predicted HLA binding affinity (left – high, middle – low, right – very low). The position of the individual epitope is depicted on the x-axis, together with the length of the respective peptide. The color intensities are described below for the respective M1 and M2 frameshift peptides. (**B**) HLA binding predictions for the additional cMS from the Top 10 IRS, including M1 frameshift peptides LTN1, MARCKS, SLC22A9, SLC35F5, MYH11, TTK, TCF7L2, CASP5 and the BANP M2 frameshift peptide are depicted for the HLA supertype representatives.


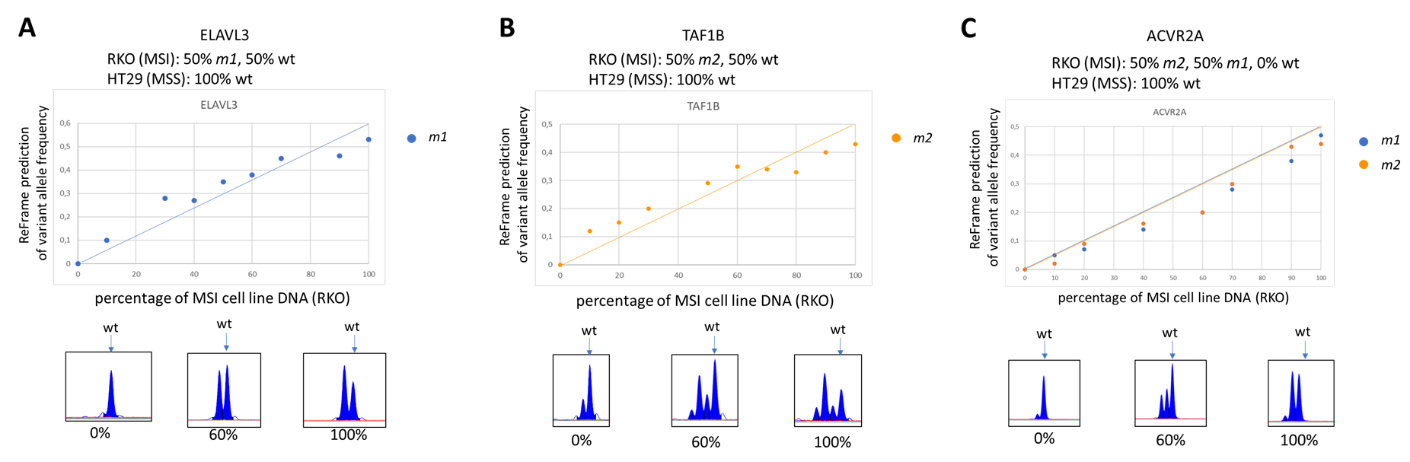


**Supplementary Figure 7. Validation of ReFrame.** MSI cell lines with known mutation status of cMS (MSI cell line RKO and microsatellite-stable cell line HT29 as a reference) were mixed to compare experimental ReFrame results (dots) with theoretically expected values (line) according to the experimental setup shown in Supplementary Figure 1D. Three scenarios were evaluated separately: *m1*wt vs. wt (A, *ELAVL3*), *m2*wt vs. wt (B, *TAF1B*), and *m2m1* vs. wt (C, *ACVR2*). Low amounts of template DNA (1 ng) were used for each PCR to simulate critical amplification conditions. Maximum deviations of measured vs. expected variant allele frequency were below 12%. The obtained results are visualized in blue for *m1* alleles and in orange for *m2* alleles. Source data are provided as a Source Data file.


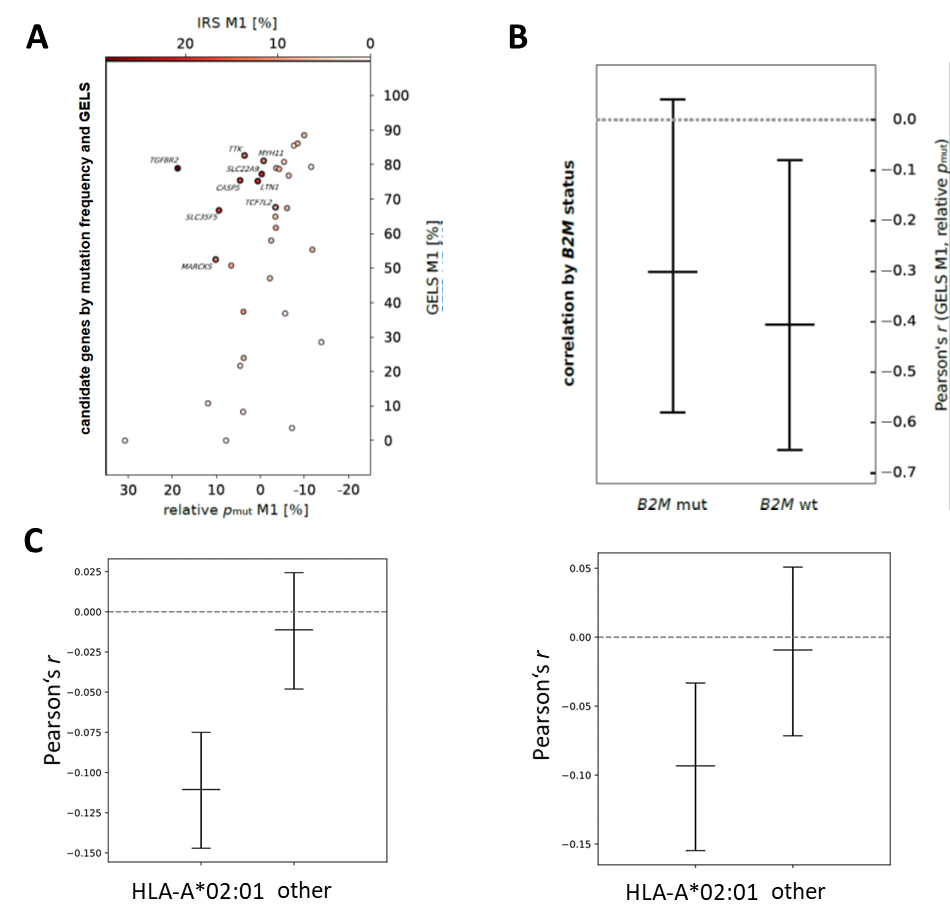


**Supplementary Figure 8. Correlation of relative mutation frequency with GELS.** Replication of correlation analyses displayed in Figures 4C, 4D, 5A, 5B using relative mutation frequency (relative *p*_mut_), computed by subtracting the mean M1 mutational frequency for cMS of a given length from the M1 mutation frequency observed for a given microsatellite. **(A)** A significant negative correlation between relative *p*_mut_ and GELS was observed. For the calculation of the GELS, all predicted epitopes (IC_50_ < 500 nM) were taken into account, with an assumed probability for a binder to be a true positive of *p*_binding_=50%. Every bubble represents one candidate. The gradient intensity of the bubbles shows the IRS, with white color representing a low IRS, while dark red displays a high IRS. All candidates with an IRS of 10% or higher are annotated. **(B)** Correlation between GELS and the relative frequency of the respective cMS mutations in MSI colorectal cancer separated by *B2M* mutation status. The Pearson´s *r* from the correlation test is shown on the y-axis, while the different groups of tumors are shown on the x-axis. Centers indicate Pearson’s *r*, Whiskers indicate 95% confidence intervals. A significant inverse correlation was observed showing *r*=-0.41, *p*=0.0147 at *n*=41 candidates for 99 MSI colorectal cancers with wild type *B2M*, with a conservative estimate of predicted epitope fidelity of *p*_binding_=50%. **(C)** Correlation between the probability of at least one putative binder truly binding to HLA-A*02:01 within an M1 frameshift peptide and the respective M1 mutation rate relative to the cMS repeat mean mutation rate in *B2M*-wildtype HLA-A*02:01-positive and -negative CRC (left) and EC (right). The Pearson´s *r* from the correlation test is shown on the y-axis, while the different groups of tumors are shown on the x-axis. Centers indicate Pearson’s *r*, whiskers indicate 95% confidence intervals. A highly significant inverse correlation was observed showing 𝑟 = −0.11, 𝑝 = 4.6 * 10^-9^ at 𝑛 = 34 HLA-A*02:01-positive CRCs with all parameters kept from Fig. 4D. For *n*=37 HLA-A*02:01-negative CRCs (other), significance did not persist at *p*=0.71. Similarly, a significant inverse correlation between HLA-A*02:01 binding scores and mutation frequency was detected for HLA-A*02:01-positive MSI EC (*n*=13, *r*=-0-09, *p*=0.003), but not for HLA-A*02:01-negative MSI EC (*n*=14).


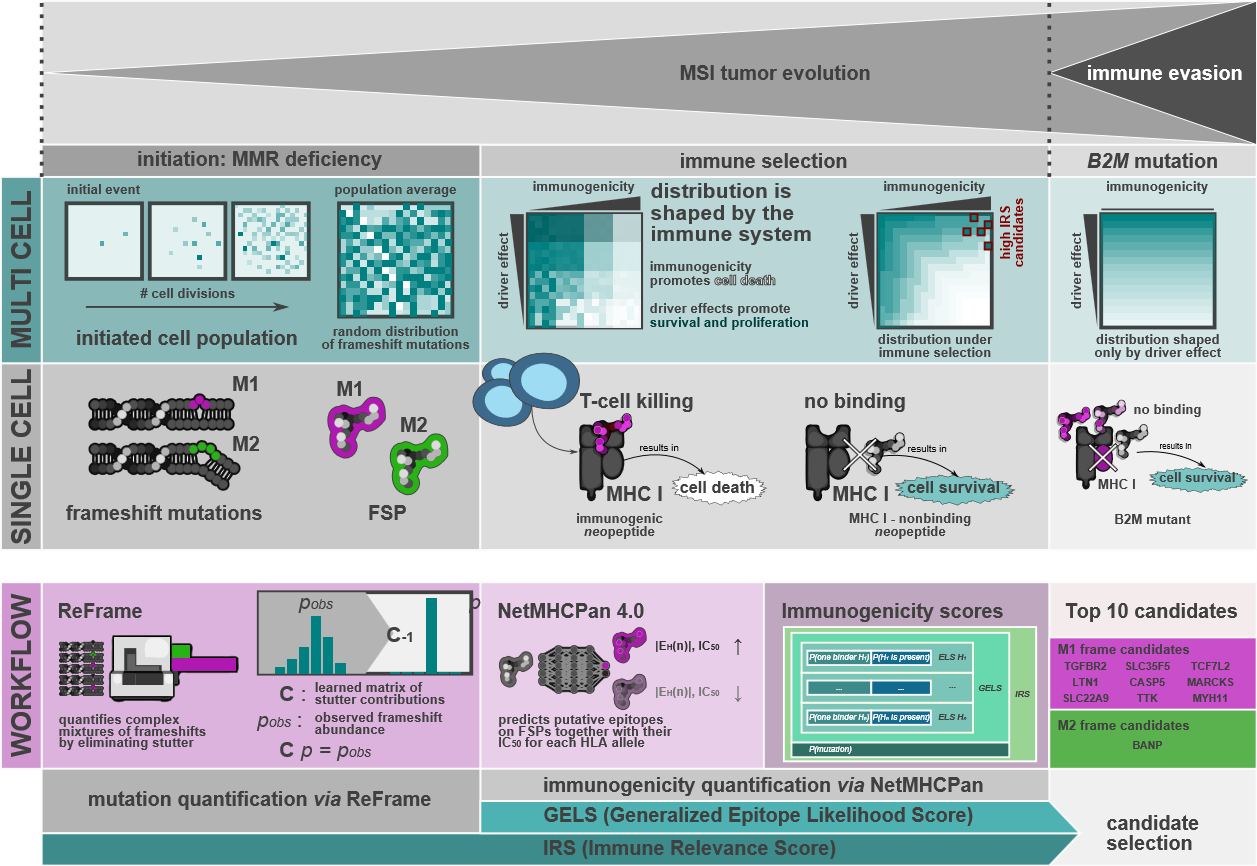


**Supplementary Figure 9. Implications of immune selection during MSI tumor evolution.** (**MULTI CELL**) Inactivation of the MMR system results in the accumulation of a high number of somatic cMS mutations during cell division. These cMS mutation events depend on the likelihood of polymerase slippage at the microsatellite loci, i.e. on microsatellite length, but are random with regard to the functional consequences of the mutations, which results in a random distribution of cMS mutations in the initiated cell population. During progression, driver mutations promoting cell survival and proliferation are favorable, while highly immunogenic mutations are unfavorable due to immune supervision. As such, the distribution of cMS mutations across a cell population is shaped by both driver effects and immune supervision. Abrogation of cellular antigen presentation, i.e. due to *B2M* mutation-induced loss of HLA class I stability, the immunogenicity of frameshift peptides resulting from cMS mutations is expected to become irrelevant for the selection of cell clones. Therefore, the distribution of cMS mutations is no longer shaped by the immune system and depends only on driver effects. (**SINGLE CELL**) Insertions and deletions due to polymerase slippage in cMS result in two equivalence classes of frameshift neopeptides with M1 or M2 frameshifts. Survival of a given cell with cMS mutations then depends on the binding affinity of these frameshift peptides to the cell’s HLA class I complexes. If frameshift peptides contain MHC ligands, they can be recognized as foreign by T cells, resulting in the possibility of T cell-mediated induction of cell death. In contrast, frameshift peptides not containing MHC ligands are neutral and do not impair cell survival. Destabilization of HLA class I by *B2M* mutation leads to a general lack of peptide-containing HLA class I complexes on the cell surface, theoretically corresponding to a complete lack of HLA class I binders. (**WORKFLOW**) Distribution of cMS mutations across tumor samples was quantified using ReFrame, which performs deconvolution on observed frameshift sequence abundances including stutter contributions to recover the true abundance of each frameshift sequence. NetMHCPan 4.0^1^ predicts the IC_50_ of putative epitopes for all cMS-derived frameshift peptides, identifying potential highly immunogenic frameshift peptides by their number of predicted low-IC_50_ epitopes. This information is composed into a hierarchy of immunogenicity scores (ELS, GELS, IRS) combining multiple probabilities of HLA class I binding, presence of correct HLA types and presence of cMS mutations. The top 10 IRS frameshift peptides are picked as possible candidates for vaccination.

**SUPPLEMENTARY TABLES**

**Supplementary Table 1. Comparison of mutation frequencies obtained by ReFrame vs literature methods**. The mutational frequencies from the cohort of analyzed cMS candidates were compared to results from previous studies. Recent comprehensive next generation sequencing (NGS) based studies or online databases, where sufficient data were available, were included. Gene name, cMS repeat length and corresponding amino acid change are listed. The studies from Hause et al. (2016)^2^, Cortes-Ciriano et al. (2017)^3^ and Kondelin et al. (2017)^4^ as well as our ReFrame algorithm are marked with an asterisk, as they show tailored approaches to enhance the detection of cMS mutations. In Kondelin et al. (2017)^4^, cMS that were not analyzed by Sanger sequencing are marked n.a.

| TU ID | MSI.Status | Tumor.type | Hereditary/sporadic | *B2M*.Seq | HLA.Type |
| --- | --- | --- | --- | --- | --- |
| HDCS0001 | MSI | CRC | hereditary | mutated | NA |
| HDCS0003 | MSI | CRC | hereditary | mutated | A02 |
| HDCS0004 | MSI | CRC | hereditary | mutated | A02 |
| HDCS0005 | MSI | CRC | hereditary | wildtype | NA |
| HDCS0006 | MSI | CRC | hereditary | wildtype | NA |
| HDCS0007 | MSI | CRC | hereditary | mutated | NA |
| HDCS0008 | MSI | CRC | hereditary | wildtype | NA |
| HDCS0009 | MSI | CRC | hereditary | mutated | NA |
| HDCS0010 | MSI | CRC | hereditary | mutated | NA |
| HDCS0011 | MSI | CRC | hereditary | NA | NA |
| HDCS0012 | MSI | CRC | hereditary | wildtype | NA |
| HDCS0013 | MSI | CRC | hereditary | wildtype | NA |
| HDCS0014 | MSI | CRC | hereditary | wildtype | NA |
| HDCS0015 | MSI | CRC | hereditary | wildtype | NA |
| HDCS0017 | MSI | CRC | hereditary | wildtype | NA |
| HDCS0022 | MSI | CRC | hereditary | wildtype | NA |
| HDCS0023 | MSI | CRC | hereditary | wildtype | NA |
| HDCS0024 | MSI | CRC | hereditary | mutated | NA |
| HDCS0025 | MSI | CRC | hereditary | wildtype | NA |
| HDCS0026 | MSI | CRC | hereditary | mutated | NA |
| HDCS0028 | MSI | CRC | hereditary | NA | NA |
| HDCS0029 | MSI | CRC | hereditary | wildtype | NA |
| HDCS0031 | MSI | CRC | hereditary | NA | NA |
| HDCS0033 | MSI | CRC | hereditary | mutated | NA |
| HDCS0035 | MSI | CRC | hereditary | wildtype | NA |
| HDCS0036 | MSI | CRC | hereditary | wildtype | Non |
| HDCS0037 | MSI | CRC | sporadic | wildtype | Non |
| HDCS0038 | MSI | CRC | sporadic | mutated | NA |
| HDCS0039 | MSI | CRC | sporadic | wildtype | NA |
| HDCS0040 | MSI | CRC | hereditary | NA | NA |
| HDCS0041 | MSI | CRC | hereditary | mutated | A02 |
| HDCS0043 | MSI | CRC | sporadic | wildtype | A02 |
| HDCS0044 | MSI | CRC | hereditary | NA | Non |
| HDCS0045 | MSI | CRC | hereditary | wildtype | Non |
| HDCS0046 | MSI | CRC | hereditary | wildtype | Non |
| HDCS0047 | MSI | CRC | hereditary | wildtype | A02 |
| HDCS0049 | MSI | CRC | sporadic | wildtype | NA |
| HDCS0050 | MSI | CRC | hereditary | wildtype | NA |
| HDCS0051 | MSI | CRC | sporadic | NA | NA |
| HDCS0052 | MSI | CRC | sporadic | wildtype | A02 |
| HDCS0054 | MSI | CRC | hereditary | wildtype | Non |
| HDCS0055 | MSI | CRC | sporadic | wildtype | A02 |
| HDCS0056 | MSI | CRC | hereditary | wildtype | A02 |
| HDCS0057 | MSI | CRC | sporadic | mutated | Non |
| HDCS0058 | MSI | CRC | sporadic | wildtype | Non |
| HDCS0059 | MSI | CRC | hereditary | mutated | Non |
| HDCS0060 | MSI | CRC | hereditary | wildtype | A02 |
| HDCS0061 | MSI | CRC | NA | wildtype | Non |
| HDCS0062 | MSI | CRC | sporadic | wildtype | NA |
| HDCS0063 | MSI | CRC | sporadic | wildtype | Non |
| HDCS0064 | MSI | CRC | sporadic | mutated | Non |
| HDCS0065 | MSI | CRC | sporadic | wildtype | NA |
| HDCS0066 | MSI | CRC | sporadic | wildtype | NA |
| HDCS0067 | MSI | CRC | sporadic | wildtype | A02 |
| HDCS0068 | MSI | CRC | hereditary | wildtype | A02 |
| HDCS0069 | MSI | CRC | sporadic | wildtype | NA |
| HDCS0070 | MSI | CRC | sporadic | wildtype | A02 |
| HDCS0071 | MSI | CRC | sporadic | wildtype | A02 |
| HDCS0072 | MSI | CRC | sporadic | mutated | A02 |
| HDCS0073 | MSI | CRC | sporadic | wildtype | A02 |
| HDCS0074 | MSI | CRC | sporadic | mutated | Non |
| HDCS0075 | MSI | CRC | sporadic | mutated | Non |
| HDCS0076 | MSI | CRC | sporadic | wildtype | NA |
| HDCS0077 | MSI | CRC | hereditary | wildtype | A02 |
| HDCS0078 | MSI | CRC | sporadic | wildtype | A02 |
| HDCS0080 | MSI | CRC | sporadic | wildtype | Non |
| HDCS0081 | MSI | CRC | sporadic | mutated | NA |
| HDCS0082 | MSI | CRC | hereditary | wildtype | Non |
| HDCS0083 | MSI | CRC | sporadic | wildtype | Non |
| HDCS0084 | MSI | CRC | sporadic | wildtype | A02 |
| HDCS0085 | MSI | CRC | sporadic | wildtype | A02 |
| HDCS0086 | MSI | CRC | hereditary | wildtype | NA |
| HDCS0087 | MSI | CRC | sporadic | mutated | Non |
| HDCS0088 | MSI | CRC | sporadic | wildtype | NA |
| HDCS0089 | MSI | CRC | hereditary | wildtype | NA |
| HDCS0090 | MSI | CRC | sporadic | wildtype | Non |
| HDCS0091 | MSI | CRC | hereditary | wildtype | Non |
| HDCS0092 | MSI | CRC | sporadic | mutated | Non |
| HDCS0093 | MSI | CRC | hereditary | wildtype | Non |
| HDCS0094 | MSI | CRC | sporadic | wildtype | A02 |
| HDCS0095 | MSI | CRC | sporadic | wildtype | Non |
| HDCS0096 | MSI | CRC | sporadic | mutated | A02 |
| HDCS0097 | MSI | CRC | sporadic | wildtype | A02 |
| HDCS0098 | MSI | CRC | hereditary | mutated | A02 |
| HDCS0099 | MSI | CRC | sporadic | wildtype | Non |
| HDCS0100 | MSI | CRC | hereditary | wildtype | NA |
| HDCS0102 | MSI | CRC | sporadic | wildtype | NA |
| HDCS0103 | MSI | CRC | sporadic | wildtype | NA |
| HDCS0104 | MSI | CRC | sporadic | wildtype | NA |
| HDCS0105 | MSI | CRC | hereditary | wildtype | Non |
| HDCS0106 | MSI | CRC | hereditary | wildtype | Non |
| HDCS0107 | MSI | CRC | hereditary | wildtype | A02 |
| HDCS0108 | MSI | CRC | sporadic | NA | A02 |
| HDCS0109 | MSI | CRC | hereditary | mutated | NA |
| HDCS0110 | MSI | CRC | sporadic | wildtype | A02 |
| HDCS0111 | MSI | CRC | sporadic | wildtype | A02 |
| HDCS0112 | MSI | CRC | hereditary | wildtype | Non |
| HDCS0113 | MSI | CRC | hereditary | wildtype | A02 |
| HDCS0114 | MSI | CRC | sporadic | wildtype | A02 |
| HDCS0115 | MSI | CRC | hereditary | mutated | NA |
| HDCS0116 | MSI | CRC | sporadic | wildtype | Non |
| HDCS0117 | MSI | CRC | sporadic | wildtype | Non |
| HDCS0118 | MSI | CRC | sporadic | wildtype | Non |
| HDCS0119 | MSI | CRC | sporadic | wildtype | A02 |
| HDCS0120 | MSI | CRC | hereditary | wildtype | Non |
| HDCS0121 | MSI | CRC | sporadic | mutated | A02 |
| HDCS0122 | MSI | CRC | hereditary | wildtype | NA |
| HDCS0123 | MSI | CRC | sporadic | mutated | A02 |
| HDCS0124 | MSI | CRC | hereditary | wildtype | A02 |
| HDCS0125 | MSI | CRC | hereditary | NA | Non |
| HDCS0126 | MSI | CRC | hereditary | wildtype | Non |
| HDCS0127 | MSI | CRC | hereditary | NA | NA |
| HDCS0128 | MSI | CRC | hereditary | wildtype | NA |
| HDCS0129 | MSI | CRC | hereditary | NA | NA |
| HDCS0130 | MSI | CRC | hereditary | wildtype | NA |
| HDCS0132 | MSI | CRC | hereditary | NA | NA |
| HDCS0133 | MSI | CRC | hereditary | wildtype | Non |
| HDCS0134 | MSI | CRC | sporadic | NA | A02 |
| HDCS0135 | MSI | CRC | hereditary | wildtype | NA |
| HDCS0136 | MSI | CRC | hereditary | wildtype | NA |
| HDCS0137 | MSI | CRC | hereditary | wildtype | NA |
| HDCS0138 | MSI | CRC | hereditary | wildtype | NA |
| HDCS0139 | MSI | CRC | hereditary | mutated | NA |
| HDCS0140 | MSI | CRC | hereditary | wildtype | Non |
| HDCS0142 | MSI | CRC | hereditary | wildtype | NA |
| HDCS0143 | MSI | CRC | hereditary | NA | Non |
| HDCS0144 | MSI | CRC | hereditary | NA | Non |
| HDCS0145 | MSI | CRC | hereditary | NA | NA |
| HDCS0148 | MSI | CRC | hereditary | wildtype | NA |
| HDCS0149 | MSI | CRC | hereditary | wildtype | NA |
| HDCS0150 | MSI | CRC | hereditary | wildtype | NA |
| HDCS0151 | MSI | CRC | hereditary | wildtype | NA |
| HDCS0152 | MSI | CRC | hereditary | NA | NA |
| HDCS0153 | MSI | CRC | hereditary | NA | NA |
| HDCS0154 | MSI | CRC | hereditary | wildtype | NA |
| HDCS0155 | MSI | CRC | hereditary | mutated | NA |
| HDCS0156 | MSI | CRC | hereditary | wildtype | NA |
| HDCS0157 | MSI | CRC | hereditary | wildtype | NA |
| HDCS0158 | MSI | CRC | hereditary | wildtype | NA |
| HDCS0159 | MSI | CRC | hereditary | wildtype | NA |
| HDCS0160 | MSI | CRC | NA | NA | NA |
| HDCS0161 | MSI | CRC | sporadic | NA | NA |
| HDES0001 | MSI | EC | NA | NA | NA |
| HDES0002 | MSI | EC | NA | NA | Non |
| HDES0003 | MSI | EC | NA | NA | Non |
| HDES0004 | MSI | EC | NA | NA | Non |
| HDES0005 | MSI | EC | NA | NA | Non |
| HDES0006 | MSI | EC | NA | NA | Non |
| HDES0007 | MSI | EC | NA | NA | Non |
| HDES0008 | MSI | EC | NA | NA | Non |
| HDES0009 | MSI | EC | NA | NA | A02 |
| HDES0010 | MSI | EC | NA | NA | A02 |
| HDES0011 | MSI | EC | NA | NA | Non |
| HDES0012 | MSI | EC | NA | NA | Non |
| HDES0013 | MSI | EC | NA | NA | Non |
| HDES0014 | MSI | EC | NA | NA | A02 |
| HDES0015 | MSI | EC | NA | NA | Non |
| HDES0016 | MSI | EC | NA | NA | Non |
| HDES0017 | MSI | EC | NA | NA | A02 |
| HDES0018 | MSI | EC | NA | NA | A02 |
| HDES0019 | MSI | EC | NA | NA | A02 |
| HDES0020 | MSI | EC | NA | NA | A02 |
| HDES0021 | MSI | EC | NA | NA | Non |
| HDES0022 | MSI | EC | NA | NA | A02 |
| HDES0023 | MSI | EC | NA | NA | A02 |
| HDES0024 | MSI | EC | NA | NA | A02 |
| HDES0025 | MSI | EC | NA | NA | A02 |
| HDES0026 | MSI | EC | NA | NA | Non |
| HDES0027 | MSI | EC | NA | NA | A02 |
| HDES0028 | MSI | EC | NA | NA | A02 |

**Supplementary Table 2. Sample cohort: Overview of sample cohort.** All tumors used in this study were MSI. Tumor type (CRC or EC), etiology (hereditary or sporadic), *B2M* sequencing status and HLA-A*02:01 status are specified.


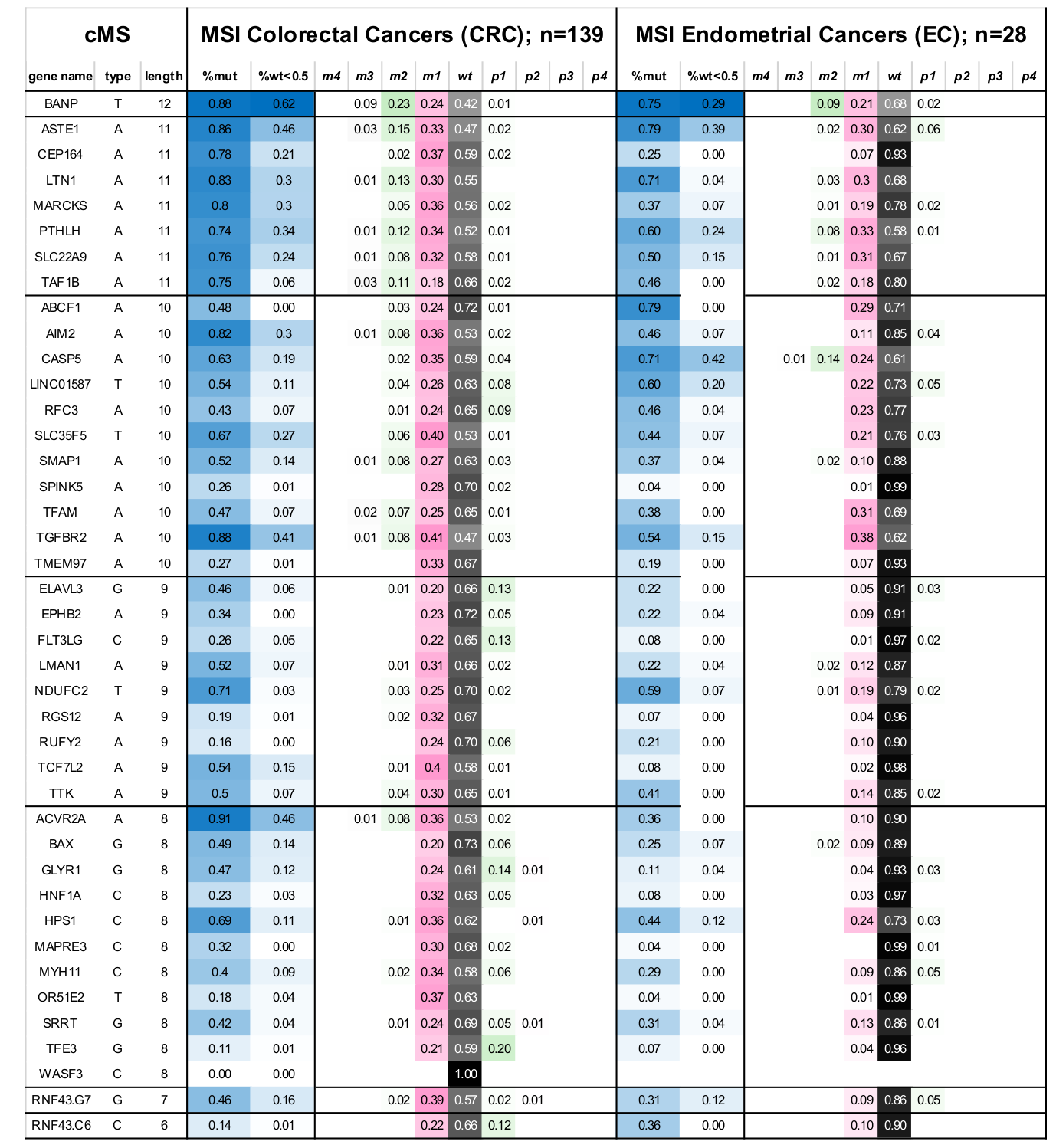


**Supplementary Table 3. Mutation frequencies and mean allele ratios resulting from ReFrame analysis.** A comprehensive overview of all analyzed cMS, showing the mutation frequencies (%mut), the ratio of samples with biallelic hits, indicated by a wt ratio <0.5 (%wt<0.5), as well as the mean mutational pattern for the cMS candidates sorted by their length. The allele ratios are depicted for wild-type (wt), minus one up to four base pair deletions (*m1* – *m4*) and plus one up to four base pair insertions (*p1* – *p4*).

| **Gene** | **Type** | **Length** | **M1>M2** | **M2>M1** | **M1EXP** | **M2EXP** | **Binomial test:** *p* **two tailed** |
| --- | --- | --- | --- | --- | --- | --- | --- |
| *ABCF1* | A | 10 | 30 | 4 | 27.04 | 6.96 | 0.29 |
| *ACVR2A* | A | 8 | 114 | 5 | 94.64 | 24.36 | < 0.0001 |
| *AIM2* | A | 10 | 90 | 18 | 85.89 | 22.11 | 0.4 |
| *ASTE1* | A | 11 | 72 | 30 | 81.12 | 20.88 | 0.04 |
| *BANP* | T | 12 | 51 | 48 | 78.73 | 20.27 | 1 |
| *BAX* | G | 8 | 28 | 8 | 28.63 | 7.37 | 0.84 |
| *CASP5* | A | 10 | 63 | 13 | 60.44 | 15.56 | 0.57 |
| *CEP164* | A | 11 | 54 | 5 | 46.92 | 12.08 | 0.02 |
| *ELAVL3* | G | 9 | 32 | 26 | 46.13 | 11.87 | < 0.0001 |
| *EPHB2* | A | 9 | 19 | 5 | 19.09 | 4.91 | 1 |
| *FLT3LG* | C | 9 | 12 | 7 | 15.11 | 3.89 | 0.09 |
| *GLYR1* | G | 8 | 38 | 19 | 45.33 | 11.67 | 0.02 |
| *HNF1A* | C | 8 | 15 | 3 | 14.31 | 3.69 | 1 |
| *HPS1* | C | 8 | 48 | 0 | 38.17 | 9.83 | < 0.0001 |
| *LINC01587* | T | 10 | 49 | 16 | 51.69 | 13.31 | 0.44 |
| *LMAN1* | A | 9 | 61 | 6 | 53.28 | 13.72 | 0.02 |
| *LTN1* | A | 11 | 79 | 32 | 88.27 | 22.73 | 0.03 |
| *MAPRE3* | C | 8 | 21 | 2 | 18.29 | 4.71 | 0.2 |
| *MARCKS* | A | 11 | 81 | 15 | 76.35 | 19.65 | 0.31 |
| *MYH11* | C | 8 | 34 | 9 | 34.2 | 8.8 | 1 |
| *NDUFC2* | T | 9 | 69 | 11 | 63.62 | 16.38 | 0.17 |
| *OR51E2* | T | 8 | 13 | 0 | 10.34 | 2.66 | 0.08 |
| *PTHLH* | A | 11 | 66 | 24 | 71.57 | 18.43 | 0.15 |
| *RFC3* | A | 10 | 33 | 16 | 38.97 | 10.03 | 0.05 |
| *RGS12* | A | 9 | 14 | 0 | 11.13 | 2.87 | 0.09 |
| *RNF43.C6* | C | 6 | 6 | 3 | 7.16 | 1.84 | 0.4 |
| *RNF43.G7* | G | 7 | 34 | 3 | 29.42 | 7.58 | 0.07 |
| *RUFY2* | A | 9 | 9 | 3 | 9.54 | 2.46 | 0.72 |
| *SLC22A9* | A | 11 | 73 | 19 | 73.16 | 18.84 | 1 |
| *SLC35F5* | T | 10 | 74 | 7 | 64.42 | 16.58 | 0.01 |
| *SMAP1* | A | 10 | 25 | 10 | 27.83 | 7.17 | 0.29 |
| *SPINK5* | A | 10 | 17 | 1 | 14.31 | 3.69 | 0.15 |
| *SRRT* | G | 8 | 24 | 6 | 23.86 | 6.14 | 1 |
| *TAF1B* | A | 11 | 52 | 40 | 73.16 | 18.84 | < 0.0001 |
| *TCF7L2* | A | 9 | 63 | 0 | 50.1 | 12.9 | < 0.0001 |
| *TFAM* | A | 10 | 32 | 11 | 34.2 | 8.8 | 0.45 |
| *TFE3* | G | 8 | 4 | 4 | 6.36 | 1.64 | 0.06 |
| *TGFBR2* | A | 10 | 80 | 16 | 76.35 | 19.65 | 0.45 |
| *TMEM97* | A | 10 | 20 | 0 | 15.91 | 4.09 | 0.02 |
| *TTK* | A | 9 | 49 | 5 | 42.94 | 11.06 | 0.04 |
| *WASF3* | C | 8 | NA | NA | NA | NA | NA |

**Supplementary Table 4. cMS mutation frames**. Distribution of cMS mutation reading frames. Corresponding cMS length, nucleotide and gene are specified. Column M1>M2 provides the numbers of tumors, for which a higher percentage of M1 compared to M2 alleles has been observed for each of the listed markers. M2>M1 provides the numbers of tumors, for which a higher percentage of M2 compared to M1 alleles has been observed. Given the actual distribution of M1 and M2 frame mutations, expected values (M1EXP and M2EXP) are compared to the observed values of M1 or M2 dominance by a binomial test. Significant differences indicate a deviation from a distribution that assumes identical probabilities for M1 and M2 mutations.

**
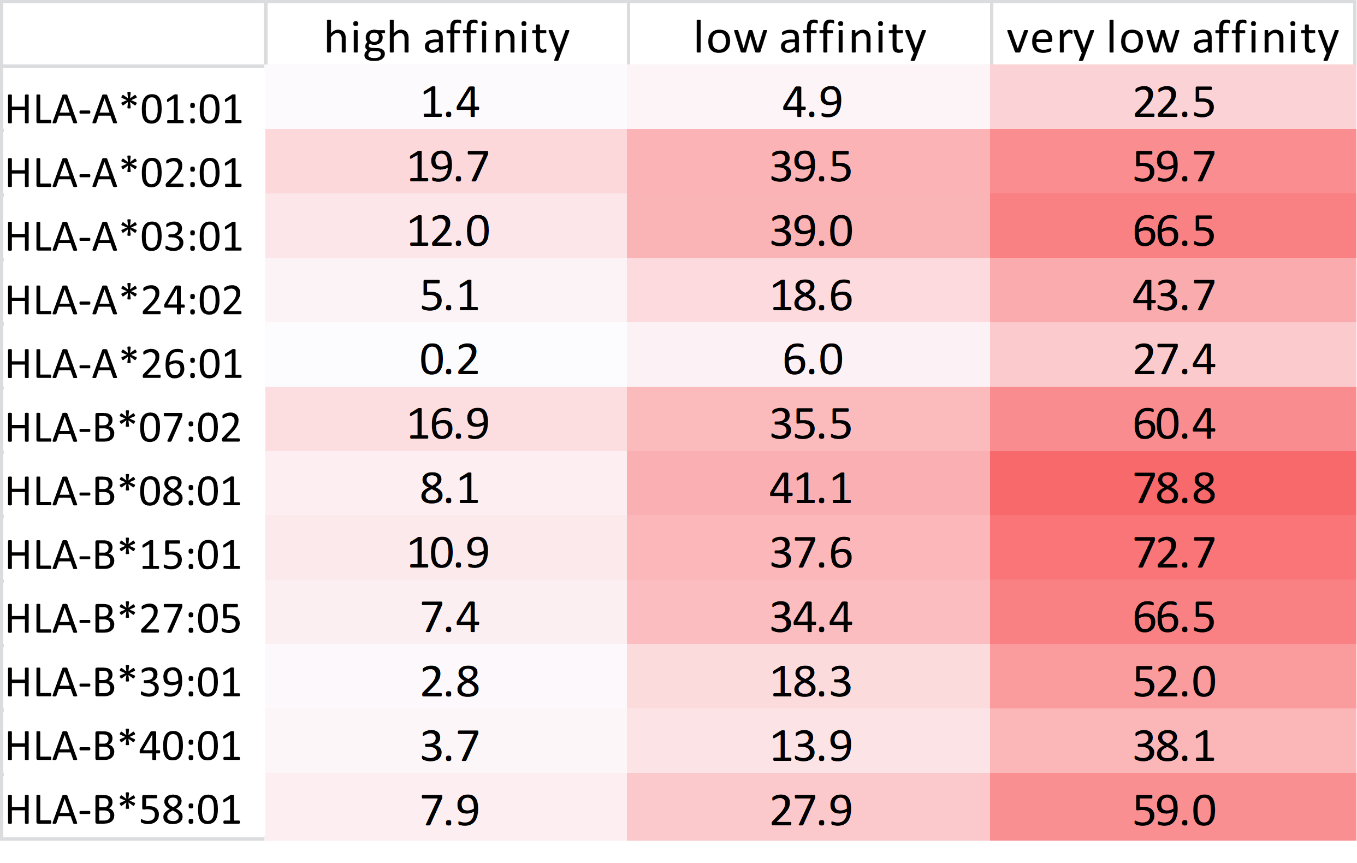
**

**Supplementary Table 5. Candidates per HLA**. For each HLA allele and each affinity class (high-affinity, low-affinity, very low-affinity), the percentage of candidates with at least one predicted ligand for the respective HLA allele is given.

| **Gene** | **cMS repeat** | **Size (bp)** | **Forward Primer [5´ > 3´]** | **Reverse Primer [5´ > 3´]** |
| --- | --- | --- | --- | --- |
| *ACVR2A* | A8 | 113 | GTTGCCATTTGAGGAGGAAA | CAGCATGTTTCTGCCAATAATC |
| *AIM2* | A10 | 76 | TTCTCCATCCAGGTTATTAAGGC | TTAGACCAGTTGGCTTGAATTG |
| *ASTE1* | A11 | 117 | ATATGCCCCCGCTGAAATA | TTGGTGTGTGCAGTGGTTCT |
| *BANP* | T12 | 126 | TTCTGTGGAAGCTCTGCCTT | TCAAGTCGCATCAGATCCAG |
| *C4orf6* | T10 | 98 | CCAGAAGCAAATTCACAAGAC | TTTTGCGTGTTCCTTCCTTC |
| *CASP5* | A10 | 141 | CAGAGTTATGTCTTAGGTGAAGG | ACCATGAAGAACATCTTTGCCCAG |
| *CLOCK* | T9 | 73 | TCATTATGTTTAATTTCAGGCTCTTG | CACATATATTATGCTTCCATCTGTCA |
| *ELAVL3* | G9 | 134 | GATGCGACCTGTTATCTCCAG | AGGTTGGTCTTGCTGTCGTC |
| *GLYR1* | G8 | 113 | GCCTCCAGAAGCTGTGACTT | ATCACCAACATCCCGTCATT |
| *LMAN1* | A9 | 114 | CACCCATGTCAGCTTTGCTA | GGAGGAATTTGAGCACTTTCA |
| *MARCKS* | A11 | 109 | GACTTCTTCGCCCAAGGC | GCCGCTCAGCTTGAAAGA |
| *MYH11* | C8 | 77 | CGGGGATTCTCTCTCTGTTTC | CTGAAGGCATGATACCTGGTG |
| *NDUFC2* | T9 | 113 | TGAATTTCAGGTTTGCATCG | AACATTTCACGGTCCCTCAC |
| *PTHLH* | A11 | 107 | TTTCACTTTCAGTACAGCACTTCTG | GAAGTAACAGGGGACTCTTAAATAATG |
| *RFC3* | A10 | 60 | TTTCTTTGTCCACAGACTCCATC | GTTACTTGCAATGGTGCTAATTTC |
| *SEC63* | A10 | 104 | AGTAAAGGACCCAAGAAAACTGC | TGCTTTTGTTTCTGTTGCTTTG |
| *SLC22A9* | A11 | 142 | GCGCCTACAGTGCCTACTCT | GCATGTGGAGCATTTCACAC |
| *SLC35F5* | T10 | 102 | TGTGGGGAAACTTACTGCAA | TCAAGTTTCAAACATCATATGCAA |
| *TAF1B* | A11 | 137 | ACCCAAATAAAAGCCCTCAAC | CTACTTAAAATTCCATTCCATGTCC |
| *TCF7L2* | A9 | 75 | GCCTCTATTCACAGATAACTC | GTTCACCTTGTATGTAGCGAA |
| *TFAM* | A10 | 204 | CTTTGGAAAAAGAAATCATGGAC | AACTATCCCACTTCTGCCTAACTG |
| *TGFBR2* | A10 | 149 | GCTGCTTCTCCAAAGTGCAT | CAGATCTCAGGTCCCACACC |
| *TTK* | A9 | 123 | TTCTTCATCCTCCAAGACTTTT | GATTTCCACAGGGATTCAAGA |
| *ZNF294* | A11 | 142 | AAGCCGAAGAGCTCATTGAA | CAGTTGTTAATTCCCAGCCTTC |
| *TMEM97* | A10 | 95 | TGTTGCGGAGCCCCTAC | AACCACCCTGTAGGCATCTC |
| *FLT3LG* | C9 | 135 | GGGATGACGTGGTGGTG | GTGATCCAGGGCTTCAGC |
| *TFE3* | G8 | 135 | CAGAGCAGCTGGACATTGAG | GAAAGTGCAGGTCCAGAAGG |
| *SMAP1* | A10 | 94 | TCAAAACTTTGGGCTGTGTTT | TAAGTGGTTTTGCCGGCTT |
| *MSH3* | A8 | 147 | AGATGTGAATCCCCTAATCAAGC | ACTCCCACAATGCCAATAAAAAT |
| *C1orf34* | G10 | 145 | AGGGACAGGATAGACTGGGG | ATCTCCCAGTCAAAATCCCA |
| *SPINK5* | A10 | 141 | TGAGGCGTTTGTTCACTTTG | TGTCATTGCTCCTTTCTCCTG |
| *EPHB2* | A9 | 141 | AACATGCAACTCAAACGACG | TTTTATCCCCCGCAAGAAC |
| *CEP 164* | A11 | 91 | GTCAACTTCTGGGGCCATTA | ACTCACCAGCGAACTTTTGG |
| *ABCF1* | A10 | 114 | GGCAGAAATACAGCAGGGG | CATCATCCACATCCTTCTTCC |
| *TCF1* | C8 | 113 | TGGCCATGGACACGTACAG | GTGGACCTTACTGGGGGAGA |
| *HPS1* | C8 | 133 | ATGTTATTACCTGTGGCCTGC | CAATACTCACTGCGGCATCT |
| *MAPRE3* | C8 | 123 | CTCTTTTCCTCTGGGCAGTTC | GGGCTGATGGAGGATTCTTC |
| *OR51E2* | T8 | 139 | TGCAGTGCTCAACAATACAGT | ACAATAGGAGTGCGAGAGGA |
| *PRDM2* | A9 | 149 | ATCCTCTCACATCTGCCCTTA | GTGATGAGTGTCCACCTTTCT |
| *RGS12* | A9 | 137 | CCGGCTTTCAAAGAGAGAAGA | ACTGGAAACTAACTGTGCATT |
| *RUFY2* | A9 | 126 | GGTTCTTCTCTTTAGGACCCC | AGTACTAACCTCAAGAGATCCCT |
| *SRRT* | G8 | 113 | GTGGCTATGAGATGCCCTATG | CTGGATAGGCAGGACATGGT |
| *WASF3* | C8 | 114 | CCCTCAACAGACCTCAGCAG | TACAAGGCATGCTGAGTTACC |

**Supplementary Table 6. Primer sequences for ReFrame analysis.** The primer sequences for each cMS analyzed by ReFrame are depicted. Both, forward and reverse primer as well as the size of the resulting PCR product are shown.

**Supplementary References**

1 Jurtz, V. *et al.* NetMHCpan-4.0: Improved Peptide-MHC Class I Interaction Predictions Integrating Eluted Ligand and Peptide Binding Affinity Data. *J Immunol* **199**, 3360-3368, doi:10.4049/jimmunol.1700893 (2017).

2 Hause, R. J., Pritchard, C. C., Shendure, J. & Salipante, S. J. Classification and characterization of microsatellite instability across 18 cancer types. *Nature Medicine* **22**, 1342-1350, doi:10.1038/nm.4191 (2016).

3 Cortes-Ciriano, I., Lee, S., Park, W. Y., Kim, T. M. & Park, P. J. A molecular portrait of microsatellite instability across multiple cancers. *Nature Communications* **8**, 15180, doi:10.1038/ncomms15180 (2017).

4 Kondelin, J. *et al.* Comprehensive Evaluation of Protein Coding Mononucleotide Microsatellites in Microsatellite-Unstable Colorectal Cancer. *Cancer Research* **77**, 4078-4088, doi:10.1158/0008-5472.CAN-17-0682 (2017).
