# Supplementary material for: The shared frameshift mutation landscape of microsatellite-unstable cancers suggests immunoediting during tumor evolution
Source: Nat Commun. 2020 Sep 21;11:4740. doi: 10.1038/s41467-020-18514-5 (PMC7506541; doi:10.1038/s41467-020-18514-5)
Supplement: Supplementary file 3 — Reporting Summary [file 41467_2020_18514_MOESM3_ESM.pdf]

nature research

Corresponding author(s): Matthias Kloor

Last updated by author(s): Jul 31, 2020

Reporting Summary

Nature Research wishes to improve the reproducibility of the work that we publish. This form provides structure for consistency and transparency in reporting. For further information on Nature Research policies, see Authors & Referees and the Editorial Policy Checklist .

Statistics

For all statistical analyses, confirm that the following items are present in the figure legend, table legend, main text, or Methods section.

n/a	Confirmed	
☐	☒	The exact sample size (*n*) for each experimental group/condition, given as a discrete number and unit of measurement
☐	☒	A statement on whether measurements were taken from distinct samples or whether the same sample was measured repeatedly
☐	☒	The statistical test(s) used AND whether they are one- or two-sided<br>*Only common tests should be described solely by name; describe more complex techniques in the Methods section.*
☐	☒	A description of all covariates tested
☐	☒	A description of any assumptions or corrections, such as tests of normality and adjustment for multiple comparisons
☐	☒	A full description of the statistical parameters including central tendency (e.g. means) or other basic estimates (e.g. regression coefficient) AND variation (e.g. standard deviation) or associated estimates of uncertainty (e.g. confidence intervals)
☐	☒	For null hypothesis testing, the test statistic (e.g. *F*, *t*, *r*) with confidence intervals, effect sizes, degrees of freedom and *P* value noted<br>*Give P values as exact values whenever suitable.*
☒	☐	For Bayesian analysis, information on the choice of priors and Markov chain Monte Carlo settings
☒	☐	For hierarchical and complex designs, identification of the appropriate level for tests and full reporting of outcomes
☐	☒	Estimates of effect sizes (e.g. Cohen's *d*, Pearson's *r*), indicating how they were calculated

Our web collection on statistics for biologists contains articles on many of the points above.

Software and code

Policy information about availability of computer code

Data collection	Primer3web version 4.0.0
Data analysis	GeneMapper™ Software version 4.0<br>R version 3.4.3<br>Python version 3.6.8<br>NetMHCpan 4.0<br>Custom software (https://github.com/atb-data/neoantigen-landscape-msi)

For manuscripts utilizing custom algorithms or software that are central to the research but not yet described in published literature, software must be made available to editors/reviewers. We strongly encourage code deposition in a community repository (e.g. GitHub). See the Nature Research guidelines for submitting code & software for further information.

Data

Policy information about availability of data

All manuscripts must include a data availability statement. This statement should provide the following information, where applicable:

- Accession codes, unique identifiers, or web links for publicly available datasets
- A list of figures that have associated raw data
- A description of any restrictions on data availability

The datasets generated during and/or analysed during the current study are available in the github repository (https://github.com/atb-data/neoantigen-landscape-msi). Coding microsatellite data used for selecting candidate targets were obtained from the publicly available database SelTarBase (Version 201307, www.seltarbase.org).

Field-specific reporting

Please select the one below that is the best fit for your research. If you are not sure, read the appropriate sections before making your selection.

☒ Life sciences ☐ Behavioural & social sciences ☐ Ecological, evolutionary & environmental sciences

For a reference copy of the document with all sections, see nature.com/documents/nr-reporting-summary-flat.pdf

Life sciences study design

All studies must disclose on these points even when the disclosure is negative.

Sample size	139 MSI colorectal cancers, 28 MSI endometrial cancers. Sample sizes were selected to obtain a comprehensive overview of frameshift mutation patterns in MSI colorectal and endometrial cancer (n>25). In order to detect expected differences of cMS mutation patterns depending on B2M mutation status and/or HLA-A*02:01 status among MSI colorectal cancers (expected effect size>10%) with a power of 80%, cohort sizes were selected to obtain a minimum group size for B2M-mutant tumors (expected B2M mutation frequency: 30%) of n=30 tumors and HLA-A*02:01-positive tumors (expected HLA-A*02:01 population frequency 50%) of n=30.
Data exclusions	cMS marker/tumor combinations not producing detectable peaks above threshold in fragment length analysis were excluded from final analysis. cMS marker/tumor combinations not producing reproducible results in replication experiments were also excluded.
Replication	All analyses were replicated twice, and in quadruplicate for reference normal DNA. Only samples with successful replication were considered in the data set.
Randomization	This is a non-interventional, exploratory study. Randomization
Blinding	Experimentators were blinded for MSI status upon pseudonymization during performing wet lab procedures. Fragment length analyses were performed and processed for all tumors in identical and automated manner. MSI status was added as annotation in the results table.

Reporting for specific materials, systems and methods

We require information from authors about some types of materials, experimental systems and methods used in many studies. Here, indicate whether each material, system or method listed is relevant to your study. If you are not sure if a list item applies to your research, read the appropriate section before selecting a response.

Materials & experimental systems

n/a	Involved in the study	
☐	☒	Antibodies
☐	☒	Eukaryotic cell lines
☒	☐	Palaeontology
☐	☒	Animals and other organisms
☐	☒	Human research participants
☒	☐	Clinical data

Methods

n/a	Involved in the study	
☒	☐	ChIP-seq
☒	☐	Flow cytometry
☒	☐	MRI-based neuroimaging

Antibodies

Antibodies used	rat anti-mouse IFNɣ antibody (clone R4-6A2, BD Bioscience), rat anti-mouse IFNɣ antibody (clone XMG1.2, BD Bioscience)
Validation	Both antibody clones have been widely used as standard tools in ELISpot analysis. For references see:<br>https://www.bdbiosciences.com/us/applications/research/t-cell-immunology/th-1-cells/intracellular-markers/cytokines-and-chemokines/mouse/purified-rat-anti-mouse-ifn--r4-6a2/p/551216<br><br>and<br><br>https://www.bdbiosciences.com/us/applications/research/t-cell-immunology/th-1-cells/intracellular-markers/cytokines-and-chemokines/mouse/apc-rat-anti-mouse-ifn--xmg12/p/554413

Eukaryotic cell lines

Policy information about cell lines

Cell line source(s)	Cell lines HT29 (human MSS colorectal cancer), LS180 (human MSI colorectal cancer), RKO (human MSI colorectal cancer) were obtained from European Collection of Cell Cultures (ECACC).
Authentication	Cell lines were authenticated using short tandem repeat profiling.
Mycoplasma contamination	All cell lines were tested negative for mycoplasma contamination.
Commonly misidentified lines (See ICLAC register)	No commonly misidentified cell lines were used.

Animals and other organisms

Policy information about studies involving animals; ARRIVE guidelines recommended for reporting animal research

Laboratory animals	For validation of immunogenicity, HLA-A*0201/HLA-DRB1*0101-transgenic mice (n=15; female, n=8; male, n=7; age between 19 and 38 weeks at first vaccination) were vaccinated.
Wild animals	The study did not involve wild animals.
Field-collected samples	The study did not involve field-collected samples.
Ethics oversight	The study was approved by Regierungspraesidium Karlsruhe (Abteilung 3 - Landwirtschaft, Ländlicher Raum, Veterinär- und Lebensmittelwesen), approval number G302/19.

Note that full information on the approval of the study protocol must also be provided in the manuscript.

Human research participants

Policy information about studies involving human research participants

Population characteristics	All tumor specimens included were MSI. 142 colorectal (female, n=74; male, n=68) and 28 endometrial cancers (female, n=28) were included. The median age was 67 (range 24-96). Tumors, for which information was available, had the following stage: MSI CRC, 26 (UICC stage I), 41 (stage II), 25 (stage III) and 6 (stage IV). MSI ECs: 13 (FIGO IA), 7 (IB), 2 (II), 1 (IIIA), 2 (IIIC), 1 (III), 1 (IVB).
Recruitment	MSI CRC and EC tumor specimens diagnosed at Heidelberg University Hospital between January 2002 and December 2017 with sufficient DNA availability were included in the study. Additional tumor specimens from Leiden University Medical Center and Helsinki University Hospital/Central Finland Central Hospital, Jyväskylä were included in the study. Sample series were largely consecutive series of tumors tested for hereditary cancer predisposition in frame of research projects. The tumor collection analyzed entirely consists of MSI cancer specimens; therefore, self-selection bias does not have any conceivable influence on the molecular profiles and statistical results.
Ethics oversight	The study was approved by the Institutional Ethics Committee, Heidelberg University Hospital.

Note that full information on the approval of the study protocol must also be provided in the manuscript.
