# Supplementary material for: The shared frameshift mutation landscape of microsatellite-unstable cancers suggests immunoediting during tumor evolution
Source: Nat Commun. 2020 Sep 21;11:4740. doi: 10.1038/s41467-020-18514-5 (PMC7506541; doi:10.1038/s41467-020-18514-5)
Supplement: Supplementary file 4 — Descriptions of Additional Supplementary Files [file 41467_2020_18514_MOESM4_ESM.pdf]

Descriptions of Additional Supplementary Files

Supplementary Data 1

Description: cMS peptide sequences. All amino acid sequences extracted from the Seltarbase for the frameshift peptides resulting from one base pair (m1) or two base pair (m2) deletions are depicted. For this purpose, only cMS with a length of 8 or more base pairs, indicated by i.e. A8, representing eight adenine base pairs, are included. For each cMS, eight wildtype amino acids are included at the N-terminus (including 8 N-terminal wt aa).

Supplementary Data 2

Description: Predicted HLA binding peptides per candidate and HLA. For each candidate neoantigen (M1 and M2 for each coding microsatellite) and each HLA allele in this study, the presence or absence of binding peptides for the three affinity classes (high-affinity, low-affinity, very low-affinity) is given, together with the frameshift peptide with the highest number of predicted MHC ligands.

Supplementary Data 3

Description: GELS and IRS in various populations. The calculations of the general epitope likelihood score (GELS) and Immune relevance score (IRS) for different patient cohorts are depicted. The scores for a US European Caucasian, a German population, a Japanese population, as well as two US Hispanic and US African American populations are calculated for high-affinity, low-affinity and very-low affinity epitopes. The calculations were implemented for each cMS peptide sequence (Supplementary Table 3) and the respective HLA types including HLA-A*01:01, HLA-A*02:01, HLA-A*03:01, HLA-A*24:02, HLA-A*26:01, HLA-B*07:02, HLA-B*08:01, HLA-B*15:01, HLA-B*27:05, HLA-B*39:01, HLA-B*40:01 and HLA-B*58:01. The different GELS and IRS columns indicate the probability pbinding, which is assumed as the probability of at least one epitope being truly presented by the HLA molecule. Importantly, values 0.0 indicate no predicted epitope for the appropriate cMS, while ”NA” represents the lack of data from the ReFrame analysis, which was only implemented for 82 peptide sequences.
